# Acknowledgement to Reviewers of *Journal of Functional Biomaterials* in 2018

**DOI:** 10.3390/jfb10010006

**Published:** 2019-01-14

**Authors:** Journal of Functional Biomaterials Editorial Office

**Affiliations:** MDPI, St. Alban-Anlage 66, 4052 Basel, Switzerland

Rigorous peer-review is the corner-stone of high-quality academic publishing. The editorial team greatly appreciates the reviewers who contributed their knowledge and expertise to the journal’s editorial process over the past 12 months. In 2018, a total of 71 papers were published in the journal, with a median time to first decision of 16 days and a median time to publication of 42 days. The editors would like to express their sincere gratitude to the following reviewers for their cooperation and dedication in 2018:

Ainsworth, Richard I.Lee, Chi H.Alhalawani, AdelLemma, SolomonApartsin, EvgenyLiao, Shu-ChuanArnaud, FrançoiseLim, Eun-KyungAxpe, EnekoLima, RuiBado, IgorLin, Dan-JaeBaino, FrancescoLiu, Cheuk LunBanach, MarcinLiu, FeiBarshtein, GregoryLode, A.Bartneck, MatthiasLodi, Matteo BrunoBeniah, GoliathLukomska-Szymanska, MonikaBerenjian, AydinMacEwan, MatthewBernardi, SaraMajor, IanBernards, MatthewMarć, Małgorzata AnnaBertazzo, SergioMarcal, HelderBerteau, Jean-PhilippeMarradi, MarcoBiedermann, ThomasMartins, AlbinoBlencowe, AntonMassera, JonathanBorkowski, LeszekMbey, Jean AimeBoyd, ChrisMeisel, Hans JoergBrooks, PeterMerolli, AntonioBuckley, M.Mills, DavidCabral Pinto, Marina M.S.Mobed-Miremadi, MaryamCametti, CesareMorouço, PedroCannavan, DaleMróz, WaldemarCao, TongyuMuller, Werner E.G.Casimiro, Maria HelenaNagy, EndreCavas-Martínez, FranciscoNelson, Fred R.T.Cecilia, Juan AntonioNicholson, John W.Chass, GregoryOkunkova, AnnaChen, Tao-HsingOzcelikkale, AltugChereddy, Kiran KumarPadamati, Ramesh BabuCicciù, MarcoPadmanabhan, JagannathCîmpean, AnişoaraPalumbo, CarlaCollins, Maurice N.Panicker, RajeshContessi Negrini, NicolaParisi, FilippoCui, HaitaoPark, Young-GukCyganowski, PiotrPatterson, JenniferDarvell, BrianPerteghella, SaraDash, BirajaPineda, EloiDatta, RupsaPistone, AlessandroDe Aza, Piedad N.Pouponneau, PierreDeng, XiaoranPrevette, Lisa E.Dias, JulianaPuigdollers, AndreuDo, Sun HeeRallis, MicaelDunne, NicholasRanzato, EliaEbara, MitsuhiroRavuri, SudheerEscobedo-Lucea, CarmenRimondini, LiaFalconi, Mario ChristianRobinson, JenniferFare, SilviaRodriguez Lozano, Francisco JavierFareed, AmberRoggendorf, HansFauzi, IlianaRonca, AlfredoFerguson, JamieRoseti, LiviaFigueiras, AnaRusso, LauraFilová, EvaSamuel-Nakamura, ChristineForner, LeopoldoSandhu, Jagdeep K.Gajjar, ChiragSantos, João M.Gantenbein, BenjaminSantulli, GaetanoGarcía-González, Carlos A.Sarma, SauravGentile, PietroScarano, AntonioGeurts, JeroenSchmidt, FranziskaGherlone, EnricoSchwan, StefanGhidelli, MatteoSebastian, AimyGiampieri, FrancescaSeetharaman, SankaranarayananGoel, PeeyushSerpooshan, VahidGonzález, SergioSerrano, Dolores R.Gordiichuk, PavloSharma, AnjaliGreenhalgh, DavidSharma, AnuragGrolli, StefanoShirwaiker, Rohan A.Guessasma, SofianeSpagnuolo, GianricoGurikov, PavelStan, GeorgeHanna, AmgadStan, Miruna SilviaHargreaves, Iain P.Steward, RobertHayakawa, TohruSubramaniam, PrasadHe, HongliangSun, MengHernández-Alcoceba, RubénSutariya, VijaykumarHihath, JoshSuzuki, JonHixon, Katherine R.Svirskis, DarrenHou, JingweiTallarico, MarcoHsieh, Chih-ChenTaruta, SeiichiHu, YangTavares, AnthonyHumelnicu, DoinaThomas, SabuHwang, YongsungTomaszewska, EwaInoue, GoTorres Lagares, DanielJanowiak, Blythe E.Trajkovski, BrankoJenkins, Samir V.Tsai, Ang-ChenJha, MithileshTsuchiya, AkiraJones, HuwTurco, GianlucaKarpukhina, NataliaVecchione, RaffaeleKathuria, HimanshuVijayavenkataraman, SanjairajKazek-Kęsik, AlicjaWang, XiaojuKevadiya, BhaveahWeber, FranzKharkar, PrathameshWeigand, AnnikaKhurshid, ZohaibWelshhans, KristyKlar, AgnesWertz, Philip W.Klimek, KatarzynaWilczek, PiotrKonka, JoannaXi, WeixianKorte, MartinXiang, ChunhuiKramer, PhillipXiang, LiminKucinska-Lipka, JustynaYang, GuangKumar, AmitYang, TaoKumar, SuneelYasin, M. NaveedKwiecień, IwonaZafar, MuhammadKwon, Tae-YubZhang, WujieKwon, Young M.


